# Readiness to Use Medicinal Marijuana in the Practices of Polish Family Physicians

**DOI:** 10.3390/jcm15072670

**Published:** 2026-04-01

**Authors:** Magdalena Florek-Łuszczki, Stanisław Lachowski, Piotr Choina, Jarosław Chmielewski, Jarogniew J. Łuszczki

**Affiliations:** 1 Department of Medical Anthropology, Institute of Rural Health, 20-090 Lublin, Poland; florek.magdalena@imw.lublin.pl (M.F.-Ł.); choina.piotr@imw.lublin.pl (P.C.); 2 Institute of Sociology, Maria Curie-Skłodowska University, 20-032 Lublin, Poland; stanislaw.lachowski@mail.umcs.pl; 3 Department of Public Health, Medical Academy of Applied and Holistic Sciences, 02-304 Warsaw, Poland; jaroslaw.chmielewski@amh.edu.pl; 4 Department of Occupational Medicine, Medical University of Lublin, 20-090 Lublin, Poland

**Keywords:** medicinal marijuana, family physicians, opinions, social determinants, questionnaire

## Abstract

**Background/Objectives**: Although therapeutic use of medicinal marijuana by patients in Poland became legal in 2017, there remains doubt among primary care physicians (PCPs) related to prescribing medicinal marijuana to their patients. In this study, we aimed to investigate the attitudes of family physicians and the systemic barriers that influence doctors’ therapeutic decisions with respect to prescribing medicinal marijuana. **Methods**: A 28-question survey was administered to a representative group of PCPs in the Lublin province of Poland. Statistical analysis of the answers of 293 (out of 301) respondents enabled us to determine the PCPs’ levels of knowledge about medicinal marijuana and their willingness to prescribe this type of therapy for their patients. **Results**: Only 32.3% of the surveyed PCPs had encountered patients who experienced symptoms associated with medicinal marijuana use. The two groups of symptoms most frequently reported by these PCPs were emotional agitation or playfulness (50.8%) and psychomotor retardation, drowsiness, and catatonia (25.4%). Only 41.0% of the surveyed PCPs perceived risks associated with prescribing medicinal marijuana to their patients, including the possibility of patients abusing medicinal marijuana, leading to addiction; sanctions from national regulatory bodies; trade in prescriptions (so-called “counterfeit prescriptions”); a lack of control over the resale of drugs by patients; and the absence of recommendations or guidelines for the use of medicinal marijuana. Our findings also demonstrate that only 5.2% of the surveyed PCPs had already prescribed medicinal marijuana in their professional practices. **Conclusions**: Limited willingness among PCPs to prescribe medicinal marijuana is primarily due to insufficient knowledge among physicians about the therapeutic effects of medicinal marijuana, its potential adverse effects, the legal framework for prescribing medications, and associated uncertainties.

## 1. Introduction

In recent years, medicinal marijuana has gained importance as an alternative therapy for patients with chronic pain, selected neurological and oncological diseases, and psychiatric disorders. The results of an analysis of articles in the PubMed database demonstrated that by the end of 1990 (31 December 1990), only 179 articles had been published on the medical use of marijuana (Medical Subject Heading: “medical marijuana”), increasing to 9712 articles by the end of 2025 (31 December 2025). The observed increase in the number of studies confirming the effectiveness of cannabinoids is leading to a shift in public perception of this form of treatment and improved safety [1,2]. Medicinal cannabis contains approximately 500 different active compounds, over 80 of which are naturally occurring cannabinoids [3]. Most research groups have focused on the effects of cannabidiol and delta-9-tetrahydrocannabinol (THC). As regards THC, it is a psychoactive substance used medically to relieve pain, improve appetite, and reduce nausea in patients undergoing chemotherapy or to treat patients with AIDS [4,5,6]. In contrast, cannabidiol is devoid of psychoactive effects; however, it possesses analgesic, anti-inflammatory, and anti-anxiety properties and is used in the treatment of cancer, epilepsy, chronic pain, inflammation, and anxiety disorders [7,8,9]. Cannabinoids are increasingly used as a complementary therapy in the treatment of pain. Evidence remains limited and inconsistent regarding the ability of cannabinoids to reduce chronic non-cancer pain by up to 30%, and they may also provide modest relief from neuropathic pain in some patients [10,11]. However, there remains insufficient evidence that their use provides relief from other types of chronic pain [12].

Moderate adverse effects of medicinal marijuana on the central nervous system and gastrointestinal system have also been reported; however, they remain rare [13,14,15,16]. Cannabinoids are recommended as second- and third-line drugs for pain management, although it should be noted that tolerance to analgesia has been reported following repeated cannabinoid use, which may result in limited long-term benefits [17,18]. Nabiximols are recommended for the treatment of muscle spasticity in patients with multiple sclerosis, spinal cord injury, or motor neuron disease when traditional methods are ineffective. These cannabinoids reduce the negative symptoms of spasticity by 20% over a period of approximately 4 weeks [19]. However, the risk of cannabis treatment discontinuation due to adverse effects, nervous system disorders, and psychiatric disorders should be considered [20]. Regulatory agencies also recommend the use of cannabinoids such as dronabinol, nabilone, levonantradol, and nabiximols in the treatment of complicated chemotherapy-induced nausea and vomiting because they have been shown to have a strong antiemetic effect in complementary treatment, significantly improving patients’ quality of life [21].

Other research groups have examined the effects of cannabinoids in improving symptoms of psychiatric disorders such as opioid addiction, schizophrenia, depression, post-traumatic stress disorder, anorexia nervosa, Tourette syndrome [22], autism spectrum disorders [23,24], and dementia [25]. The scarcity of detailed research and the lack of data prevent regulatory agencies from considering the therapeutic use of cannabinoids in the treatment of psychiatric disorders [26]. Research on the role of the endocannabinoid system and its influence on inflammation raises hope for the use of cannabinoids in the treatment of autoimmune diseases such as rheumatoid arthritis and inflammatory bowel disease [27].

With the growing number of studies confirming the efficacy and safety of medicinal marijuana in specific therapeutic indications, an increasing number of countries—including Poland—are introducing regulations enabling its use in clinical practice. Pursuant to the Act amending the Act on Counteracting Drug Addiction of 7 July 2017, and the Act on the Reimbursement of Medicines, Foodstuffs for Particular Nutritional Uses, and Medical Devices [28], since 1 November 2017, specialists and primary care physicians (PCPs) have been able to prescribe approved medications containing cannabinoids. In Poland, any form of cannabis preparation is legally permitted provided it is prescribed by a physician and the prescription is filled in a pharmacy. Detailed conditions for issuing prescriptions for preparations containing narcotic drugs or psychoactive substances, in addition to detailed conditions for storing these substances by pharmacies, are regulated by the Regulation of the Minister of Health of 14 March 2024 [29]. The doctor prescribing medical marijuana must make a note in the patient’s file regarding the issuance of an “Rpw” (prescription for narcotic substances). As medical marijuana is not reimbursed by the Polish national health insurance system (NFZ), the patient must pay the full, high cost of the medication [30,31].

Detailed requirements and necessary legal acts regarding standardized forms of medicinal marijuana, extraction methods, dosage, and monitoring of potential adverse effects were compiled in synthetic form and announced by the Minister of Health in an official statement on 25 February 2025 [32]. However, the mere availability of cannabis-based preparations is only the beginning. Family physicians are a key link in the implementation of medicinal marijuana therapy—as the first point of contact in the healthcare system, they have the potential to initiate discussions about this form of treatment, assess its validity, and refer patients for further specialist consultations. However, are family doctors ready for such responsibility? What are their competencies, beliefs, and concerns regarding medicinal marijuana? Does the healthcare system support them in gaining knowledge and making therapeutic decisions in this area? Amid dynamic legislative changes, increasing patient demand, and ambiguous media messages, the question of family doctors’ willingness to use medicinal marijuana is becoming not only timely but also urgent.

In this article, we attempt to answer these questions by analyzing the current state of knowledge, the attitudes of family physicians, and the systemic barriers that influence their therapeutic decisions. We will also examine possible scenarios for the development of this form of therapy in primary care practice and recommendations that could support physicians in the safe and informed implementation of cannabis treatment.

## 2. Materials and Methods

This retrospective study was conducted between 2020 and 2022 among physicians working in primary healthcare facilities in the Lublin Voivodeship, where approximately 1000 physicians are employed. A total of 301 physicians participated in the study; however, the responses of 293 physicians were included in the final statistical analysis. Due to the study sample size of 278 physicians (with a confidence level of α = 0.95), the total number of 293 physicians is sufficient to ensure representativeness of the population of PCPs in the Lublin Voivodeship. It should be emphasized that despite the study being conducted within the period indicated above, the obtained results remain valid as the regulations regarding permits to prescribe medicinal cannabis in Poland have not changed to date.

To achieve our aim of assessing the willingness to use medicinal marijuana in family physician practice, four aspects of this issue were considered: (1) an assessment of the extent of contact between the surveyed physicians and symptoms suggestive of medicinal marijuana use; (2) an assessment of the frequency of prescribing medicinal marijuana by the surveyed physicians; (3) the surveyed physicians’ perception of the risks associated with prescribing medicinal marijuana; and (4) barriers preventing the surveyed physicians from prescribing medicinal marijuana. Individual dimensions of the discussed issue were analyzed based on two groups of variables: the demographic characteristics of the respondents and selected indicators of their knowledge about medicinal marijuana. The socio-demographic variables included characteristics such as gender, age, and place of residence.

The retrospective study was conducted using a diagnostic survey with a 28-item questionnaire. The survey was developed specifically for this study. Two experienced researchers, a sociologist and a physician, whose research interests include the use of cannabinoids in patient treatment, were involved in developing the survey tool. The internal consistency of the tool was assessed using the Cronbach’s alpha test and was satisfactory with an α ≥ 0.8.

Due to constraints imposed by the COVID-19 pandemic, which coincided with the study period, the research was conducted initially using an audience survey, and from the second quarter of 2020, using an online survey, adhering to the CHERRIES recommendations [33]. The study was conducted as part of a research project funded by a grant from the Ministry of Science and Higher Education entitled “Attitudes of Primary Care Physicians Toward the Use of Medicinal Cannabis in the Treatment of Patients.” The project received approval from the Bioethics Committee of the Institute of Rural Health (IMW), Decision No. 6/2019.

Both the chi-square test for independent variables (categorical) and logistic regression were performed using IBM SPSS ver. 29.0 for Windows. All figures were created in GraphPad Prism ver. 7.0 for Windows.

## 3. Results

The majority of respondents (64.2%) were women (Table 1). Considering the age of the respondents, half were young doctors—up to 30 years old—while the remaining (50.0%) were 31 years old or older (Table 1). The vast majority of physicians practiced in primary healthcare settings located in urban areas (74.8%), with the remainder practicing in rural areas or in two locations—a rural area and an urban area (Table 1). Slightly more than 50% of surveyed physicians had less than 5 years of experience, while the remaining respondents had worked in the medicinal profession for more than 5 years. More than half of the physicians (58.8%) employed in primary healthcare specialized exclusively in family medicine, while the remainder specialized in other specialties (e.g., pediatrics, neurology, diabetology, and pulmonology).

The vast majority of surveyed physicians (67.7%) had not encountered patients who experienced symptoms associated with medicinal marijuana use. The remaining 32.3% had encountered such symptoms. Physicians’ specialization in family medicine and their level of knowledge about this drug are significantly associated with observing symptoms associated with medicinal marijuana use (Table 2). Physicians with specialties other than family medicine were significantly more likely to observe symptoms of marijuana use (42.3%) than family medicine specialists (26.9%) (difference at *p* < 0.05). Physicians who rated their knowledge of this drug higher (50.0%) observed symptoms of marijuana use significantly more frequently (*p* < 0.001) than those who rated their knowledge lower (22.8%) (Table 2).

**Figure 1:**
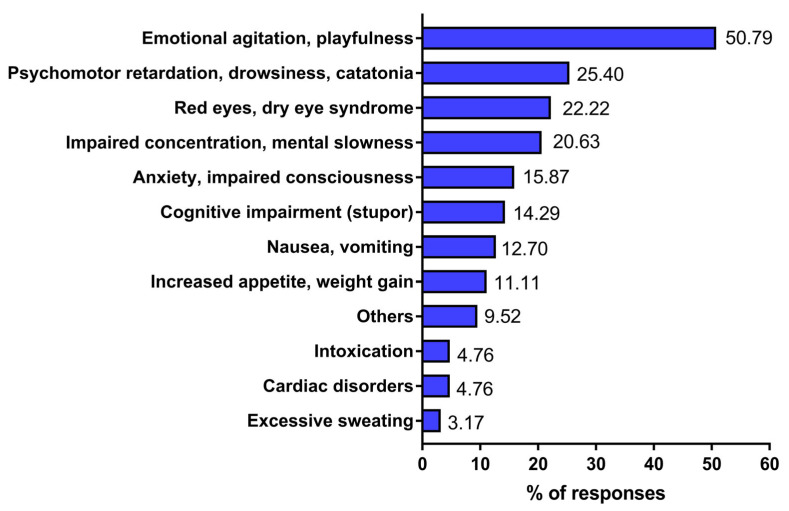
Symptoms indicating the use of medicinal marijuana encountered by the examined physician. Responses of physicians were gathered from a diagnostic survey (audience and online questionnaire) conducted between 2020 and 2022. A total of 293 physicians participated in the survey; however, only 63 out of 195 physicians reported symptoms indicating the use of medicinal marijuana by their patients. The percentage did not total 100% because respondents could provide more than one answer.

Among respondents who observed symptoms associated with medicinal marijuana use by patients, the majority reported emotional agitation or playfulness (50.8%) (Figure 1). A significantly smaller proportion of physicians observed the following in patients using marijuana: psychomotor retardation, drowsiness, and catatonia (25.4%); red eyes or dry eye syndrome (22.2%); and impaired concentration and mental slowness (20.6%). Other symptoms reported by respondents included anxiety and impaired consciousness (15.9%), cognitive impairment (14.3%), and nausea and vomiting (11.1%). A small number of individuals reported symptoms of intoxication, cardiac disorders, or excessive sweating (Figure 1).

The results of bivariate analyses do not account for the simultaneous influence of other variables; therefore, some of the identified relationships may be spurious. Multivariate logistic regression analysis was conducted to model the determinants of physicians’ practice of prescribing medicinal marijuana to their patients (Table 3). This method also enables estimation of the odds ratio (OR) of the analyzed phenomenon.

In the theoretical model, the dependent variable was a dichotomous variable—prescribing medicinal marijuana (0—no: not-prescribing, 1—yes: prescribing). Eight independent variables were introduced into the model: two quantitative variables (age and years of experience in the medical profession) and six dichotomous variables: gender (0—female, 1—male), rural location of medical practice (0—no, 1—yes), specialization in family medicine (0—no, 1—yes), belief in having the authority to prescribe medicinal marijuana (0—no, 1—yes), high self-assessment of knowledge about medicinal marijuana (0—no, 1—yes), and exposure to symptoms indicating marijuana use by patients (0—no, 1—yes). Analysis of the correlation matrix of independent variables revealed a very strong correlation between the age of the respondents and their length of service as a physician (r = 0.99). Therefore, to avoid multicollinearity, only one of these variables—the length of service as a physician (experience in the medical profession)—was entered into the model (Table 3).

Logistic regression analysis was conducted using the Wald backward elimination method. In the third stage of the analysis, the model retained predictors significantly associated with the practice of prescribing medicinal marijuana: physician experience, rural practice, belief in having the authority to prescribe marijuana, high self-assessed knowledge of marijuana, and exposure to symptoms suggestive of marijuana use by patients (Table 3). However, no statistically significant correlations were found for physician gender or specialization in family medicine. The likelihood of prescribing medicinal marijuana increases with a physician’s length of service—each additional year of service increases the odds of prescribing by approximately 3% (OR = 1.03). This likelihood is also significantly higher for physicians who believe they have the authority to prescribe marijuana (OR = 4.223—an increase in probability of nearly 422%) and for physicians who rate their knowledge of this form of therapy as high (OR = 2.328—an increase in probability of nearly 233%). In turn, the likelihood of prescribing medicinal marijuana is lower among physicians practicing in rural areas (OR = 0.475—a 52.5% lower probability) and among physicians who have encountered symptoms suggesting marijuana use in their patients (OR ≈ 0.45—a 55% lower probability) (Table 3). The obtained model was ultimately statistically significant (χ^2^ = 52.426; *p* < 0.001) and well-fitted to the data (Hosmer–Lemeshow test: χ^2^ = 2.388; *p* = 0.967). The model explains approximately 21% of the variability in the dependent variable (Nagelkerke’s R^2^ = 0.221).

**Figure 2:**
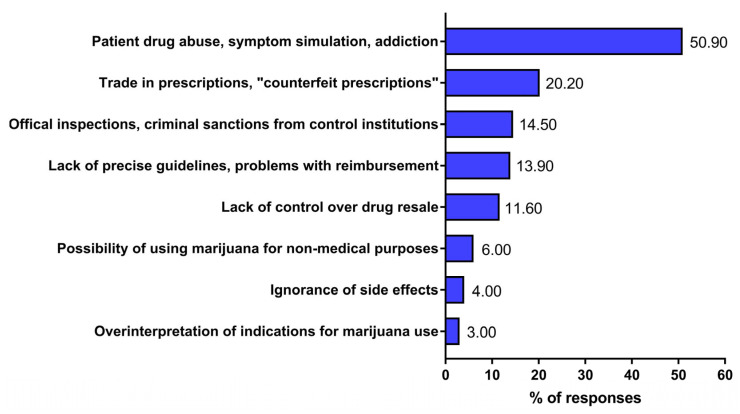
Risks associated with issuing prescriptions for medicinal marijuana to patients based on physician responses. The responses of physicians were gathered from a diagnostic survey (audience and online questionnaires) conducted between 2020 and 2022. A total of 293 physicians participated in the survey; however, only 173 perceived the risks of prescribing medicinal marijuana to patients. The percentage did not total 100% because respondents could provide more than one answer.

Among respondents who believe that prescribing medicinal marijuana to patients carries various risks, half (50.9%) point to the possibility of patients abusing the medication, which can lead to addiction (Figure 2). A significant percentage of respondents point to irregularities in the distribution of this drug, such as the trade in prescriptions (issuing so-called “counterfeit prescriptions”) (20.2%) and the lack of control over the resale of drugs (11.6%) (Figure 2). Another group of threats concerns the liability of physicians who prescribe medicinal marijuana, including criminal sanctions from regulatory bodies (the National Health Fund) (14.5%) and the lack of guidelines for the use of this medication (13.9%). A relatively small percentage of respondents perceive the risk of using the prescribed medication for purposes other than therapeutic use (6.8%), in addition to prescribing it based on over-interpretation of the indications (3.0%) or ignorance of the side effects (4.0%).

Our findings demonstrated that only 5.2% of primary care physicians have prescribed medicinal marijuana in their professional practice to date. This finding is quite similar to that observed recently in an online survey, in which only 8% physicians in Poland (general practitioners, internal medicine, and oncologists) have ever issued a prescription of medical cannabis [30].

The majority of surveyed physicians (59.0%) perceived risks associated with prescribing medicinal marijuana to patients, while the remainder of participants (41.0%) did not perceive the existence of such risks. Perception of the risks associated with prescribing medicinal marijuana was significantly associated with the practice of prescribing it and with the assessment of its availability. Among physicians who reported not prescribing medicinal marijuana, the vast majority (66.9%) perceive negative consequences of this practice. Among the remaining respondents (those who prescribed or did not answer this question), less than half of the physicians (47.9%) indicated negative consequences of its use (Table 4). Similar differences were found when analyzing the perception of risks associated with prescribing marijuana versus the opinion that access to this drug should be easier. Among physicians who disagreed with this view, a significantly higher percentage perceived risks associated with prescribing medicinal marijuana (68.8%) than among physicians who shared this view (50%).

Analysis of respondents’ opinions regarding the risks associated with prescribing medicinal marijuana indicates that some physicians refrain from writing prescriptions for this type of medication due to various limitations. This attitude was expressed by 39.4% of respondents, while the remaining 60.6% did not experience such limitations. The only factor differentiating attitudes in this regard was holding a specific specialization (Table 4).

The existence of limitations that prevent physicians from prescribing medicinal marijuana is more frequently reported by physicians specializing in medical disciplines other than family medicine (49.5%) than by family medicine specialists (32.7%). This difference is statistically significant at *p* < 0.01 (Table 5).

**Figure 3:**
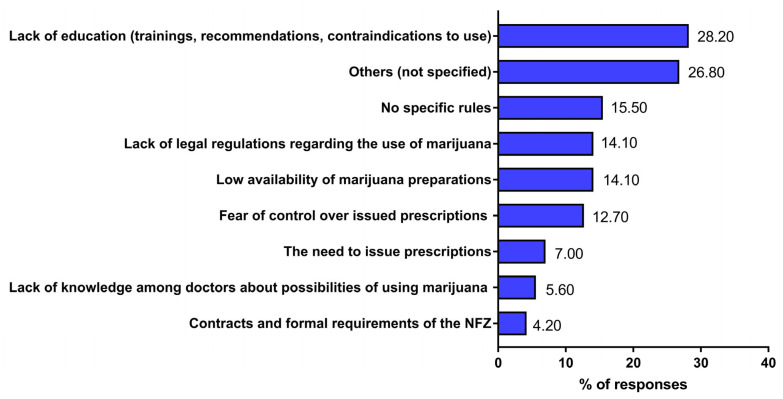
Perceived limitations on the use of medicinal marijuana among respondents. The responses of physicians were gathered from a diagnostic survey (audience and online questionnaire) conducted between 2020 and 2022. A total of 293 physicians participated in the survey; however, only 97 out of 246 physicians reported perceiving limitations on the use of medicinal marijuana for patients. The percentage did not total 100% because respondents could provide more than one answer.

Among respondents who do not prescribe medicinal marijuana due to various limitations, the majority indicate inadequate knowledge about this type of treatment. Nearly one-third (28.2%) perceive deficiencies in physician education (including a lack of training or lack of materials regarding recommendations and contraindications), while 5.6% believe that physicians generally lack sufficient knowledge about the potential use of marijuana in patients’ treatment, which constitutes a barrier to its use (Figure 3).

A relatively large group of respondents also cited the lack of legal regulations regarding its use (14.1%) and the lack of clear rules for its use (15.5%) as limitations to the use of medicinal marijuana. A portion of respondents also cited bureaucratic constraints, such as the need to issue prescriptions (7.0%), and the structure of contracts and formal requirements of the National Health Fund and the Ministry of Health (4.2%) (Figure 3).

## 4. Discussion

The ability of patients to use medicinal marijuana, beyond its therapeutic indications, largely depends on physicians’ knowledge of the drug and their willingness to prescribe it. Medicinal marijuana has been available in Poland for nine years and can be prescribed by both clinicians and primary care physicians. Given their frequent patient contact and essential role in the diagnostic and therapeutic process, including collaboration with specialists, primary care physicians’ expertise in medicinal marijuana treatment is vital, yet it also raises numerous concerns among physicians, which may lead to abandonment of this type of therapy. It is also worth emphasizing that the decision to undergo medicinal marijuana treatment is linked to obtaining the patient’s approval for its use.

The results of a cross-sectional study conducted in 2025 on a representative sample of adult Polish residents (aged 18–84), examining opinions on the legalization of medicinal marijuana, willingness to undergo cannabinoid-based therapy, and perceptions of physician and patient knowledge, demonstrated that approval for this treatment method is high. The vast majority of respondents supported the legalization of medicinal marijuana (81.1%) and expressed a willingness to undergo treatment if medically indicated (84.3%). However, only 4.2% reported receiving a medicinal marijuana recommendation from a doctor [31]. Practically identical results were obtained by Hordowicz et al., who reported that most of the surveyed physicians (93.1%, i.e., 161 out of 173) require strict guidelines for prescribing medicinal marijuana to their patients [30]. Lack of evidence-based medicine studies on therapeutic dosing of medicinal marijuana in patients was emphasized by the physicians [34]. Moreover, in a survey of general practitioners’ patients on New Zealand’s North Island, conducted between November 2018 and October 2019, 91% of patients reported that they would use a prescribed cannabis-based medicinal product, with 45% reporting that they believed it could provide some benefit to their health. However, of those who found it beneficial, fewer than 10% had discussed it with their doctor [35].

It should be emphasized that primary care physicians have varying experiences with their patients’ use of medicinal marijuana. One in three physicians surveyed reported encountering patients treated with marijuana. Physicians working in primary care with specialties other than family medicine reported significantly more contact with the use of medicinal marijuana.

In this study, 41.0% of physicians recognize the risks associated with the use of medicinal marijuana. Identifying the risks associated with prescribing marijuana is significantly related to prescribing practices and the assessment of the drug’s availability. Such risks were significantly more frequently reported by physicians who had never implemented this therapy in their practice and those who opposed easier access to medicinal marijuana treatment. Skeptics of medicinal marijuana therapy primarily cited the potential for patient abuse, which can lead to addiction, irregularities in the drug’s distribution, such as prescription trading, and a lack of control over drug resale. Family physicians in Israel perceived similar threats. Approximately 95% of physicians surveyed stated that the main barrier to recommending medicinal marijuana treatment is its potential for abuse, while 66% cited the risk of unauthorized distribution to the general public [36].

In turn, the results of a study conducted among Australian general practitioners demonstrated that the majority had either favorable or neutral opinions about the use of medicinal marijuana, with over half (56.5%) supporting the availability of medicinal marijuana by prescription. The leading indications for marijuana use, in their opinion, were cancer pain management, palliative care, and epilepsy [37,38].

The results presented herein indicate that as many as 94.8% of primary care physicians have not prescribed medicinal marijuana in their current practice. Among respondents who do not prescribe medicinal marijuana due to various limitations (39.4%), the majority cited inadequate knowledge about this type of treatment, a lack of legal regulations regarding its use, and a lack of clear guidelines for its use. Physicians’ previous experience with medicinal marijuana, perceived risks, and associated limitations may be significant factors influencing their willingness to prescribe marijuana for therapeutic purposes. The results of a comprehensive, systematic literature review demonstrated that although the majority of study participants recruited from Europe supported the use of medicinal cannabis for therapeutic reasons, they also indicated the need for additional training on the medical uses of cannabinoids and a lack of awareness of the legal status and regulations regarding medicinal cannabis among both physicians and future physicians, in addition to students of other medicinal professions (e.g., nursing and pharmacist) [39].

Similarly, the results of a study conducted among primary care physicians in Israel showed that although half of physicians expressed willingness to prescribe medicinal marijuana for various diseases, and 61% to dying patients, simultaneously, as many as 63% responded that their knowledge in this area was limited, with ¾ of the respondents declaring their willingness to broaden their knowledge and enhance their expertise [40].

Although the Lebanese parliament legalized marijuana for medical and industrial use in 2020, this decision led to varying attitudes among healthcare professionals toward its use. Only 18% of participants described their level of knowledge about the indications for prescribing medicinal marijuana to patients as good, one in four physicians surveyed expressed willingness to prescribe it, and another 30% said they “might consider” prescribing this medication for the patients [41].

In summary, the results presented herein indicate limited willingness among primary care physicians to prescribe medicinal marijuana, primarily due to insufficient knowledge among physicians about the therapeutic effects of medicinal marijuana, its potential adverse effects, the legal framework for prescribing medications, and the associated uncertainties. It is important to note that the problem of limited knowledge about the therapeutic effects of medicinal marijuana and concerns about administering it to patients is not limited to doctors working in Poland [30,31]. It is also being noted among doctors working in other countries, such as the United States, Israel, Canada, Australia, Ireland, and Norway. This commonality points to the need for educational initiatives among medical students, in addition to professionally active physicians [36,40,42,43,44,45,46,47,48].

Although our study comprised only a moderately large sample of physicians (n = 293), the main limitation of the study is the number of participants, which, while representative of the Lublin Voivodeship, is not representative of the general population of primary care physicians in Poland. Selection bias may have also influenced the online study findings, whereby the answers of those willing and able to participate and complete the survey in this manner may differ from those who declined.

## 5. Conclusions

Research findings indicate that family physicians, despite their authority to implement medicinal marijuana in patient care, have expressed a number of concerns about its use, which may reduce their willingness to prescribe it. Consequently, although public support for medicinal marijuana in Poland is high, its implementation in outpatient primary care physicians’ practices remains limited. Bridging the gap between patient expectations and physician readiness will require comprehensive educational initiatives, evidence-based guidelines, and a clear legal framework that supports healthcare professionals and informs patients.

## Figures and Tables

**Table 1:** Characteristics of the surveyed physicians.

Determinants		*n*	%
Gender	Female	188	64.2
	Male	105	35.8
	Total	293	100
Permanent residence	Urban	204	71.8
	Rural	79	28.2
	Total	280	100
Age	Up to 30 years old	143	50
	31 years old or older	143	50
	Total	286	100
Experience as a physician	Up to 5 years	160	54.8
	6 or more years	132	45.2
	Total	292	100
Place of medical practice	City	219	74.8
	Village	74	24.2
	Total	293	100
Specializations held	Family medicine only	164	58.8
	Family medicine and others	115	41.2
	Total	279	100

Responses of physicians were gathered from a diagnostic survey (audience and online questionnaire) collected between 2020 and 2022. The total number of physicians participating in this survey was 293; however, missing data were not taken into account in the analyses.

**Table 2:** Exposure of the surveyed physicians to symptoms indicating the use of medicinal marijuana according to their specialization, level of knowledge about marijuana (self-assessment).

Independent Variables	Exposure to Symptoms			
**Specialization**	Exposure	No Exposure	Total	
Family medicine	*n* = 32 (26.9%)	*n* = 87 (73.1%)	*n* = 119 (100%)	χ^2^ = 4.774
Other specialization	*n* = 30 (42.3%)	*n* = 41 (57.7%)	*n* = 71 (100%)	*p* = 0.029
**Level of knowledge about marijuana**	Exposure	No Exposure	Total	
High or moderate	*n* = 34 (50%)	*n* = 34 (50%)	*n* = 68 (100%)	χ^2^ = 14.944
Low or very low	*n* = 29 (42.3%)	*n* = 98 (57.7%)	*n* = 127 (100%)	*p* < 0.001

**Table 3:** A model of determinants of physicians’ prescription of medicinal marijuana.

Predictor (Coding)	B	SE	W	df	OR	*p*
Experience in the medical profession	0.029	0.01	7.937	1	1.03	0.005
Rural setting as a medical practice (0 no; 1 yes)	−0.745	0.287	6.72	1	0.475	0.01
Conviction of having the authority to prescribe marijuana (0 no; 1 yes)	1.44	0.368	15.347	1	4.223	<0.001
High self-assessment of knowledge about marijuana (0 no; 1 yes)	0.845	0.29	8.473	1	2.328	0.004
Doctor encountered symptoms suggesting marijuana use (0 no; 1 yes)	−0.798	0.271	8.691	1	0.45	0.003
Constant	0.922	0.468	3.879	1	2.515	0.049

Statistical analysis of data was performed with logistic regression using the backward stepwise selection technique based on the Wald test. B—unstandardized regression coefficient*;* SE—standard errors; W—Wald statistics; df—degree of freedom; OR—odds ratio; *p*—significance.

**Table 4:** Perception of risks associated with issuing prescriptions for medicinal marijuana according to medicinal marijuana prescribing practices and assessment of its availability.

Independent Variables	Spotting Threats			
**Prescribing marijuana**	Notice	Do not notice	Total	
Prescribing	*n* = 58 (47.9%)	*n* = 63 (52.1%)	*n* = 121 (100%)	χ^2^ = 10.522
Non-prescribing	*n* = 115 (66.9%)	*n* = 57 (33.1%)	*n* = 172 (100%)	*p* < 0.001
**Easier availability of marijuana**	Notice	Do not notice	Total	
Yes	*n* = 76 (50.0%)	*n* = 76 (50.0%)	*n* = 151 (100%)	χ^2^ = 10.685
No	*n* = 97 (68.3%)	*n* = 45 (31.7%)	*n* = 142 (100%)	*p* < 0.001

**Table 5:** Existence of limitations that prevent physicians from prescribing medicinal marijuana by specialty.

Independent Variables	Limitations			
**Specialization**	Occur	Do not occur	Total	
Family medicine	*n* = 48 (32.7%)	*n* = 99 (67.3%)	*n* = 147 (100%)	χ^2^ = 7.062
Other specialization	*n* = 49 (49.5%)	*n* = 50 (50.5%)	*n* = 99 (100%)	*p* = 0.008

## Data Availability

The raw data supporting the conclusions of this article will be made available by the authors upon request.

## Abbreviations

The following abbreviations are used in this manuscript:

PCPs	Primary Care Physicians

## References

[1] Hossain M.K., Chae H.J. (2024). Medical cannabis: From research breakthroughs to shifting public perceptions and ensuring safe use. Integr. Med. Res..

[2] Chhabra M., Paul A., Abulannaz O., Lê M.L., Mansell H., Finkelstein Y., Huntsman R.J., Kelly L.E. (2025). Cannabinoids for Medical Purposes in Children: A Living Systematic Review. Acta Paediatr..

[3] Aizpurua-Olaizola O., Soydaner U., Öztürk E., Schibano D., Simsir Y., Navarro P., Etxebarria N., Usobiaga A. (2016). Evolution of the Cannabinoid and Terpene Content during the Growth of *Cannabis sativa* Plants from Different Chemotypes. J. Nat. Prod..

[4] Abrams D.I. (2022). Cannabis, Cannabinoids and Cannabis-Based Medicines in Cancer Care. Integr. Cancer Ther..

[5] Mortimer T.L., Mabin T., Engelbrecht A.M. (2019). Cannabinoids: The lows and the highs of chemotherapy-induced nausea and vomiting. Future Oncol..

[6] National Academies of Sciences, Engineering, and Medicine (2017). The Health Effects of Cannabis and Cannabinoids: The Current State of Evidence and Recommendations for Research.

[7] Sen P., Sadat S., Ebisumoto K., Al-Msari R., Miyauchi S., Roy S., Mohammadzadeh P., Lips K., Nakagawa T., Saddawi-Konefka R. (2025). CBD promotes antitumor activity by modulating tumor immune microenvironment in HPV associated head and neck squamous cell carcinoma. Front. Immunol..

[8] Fiani B., Sarhadi K.J., Soula M., Zafar A., Quadri S.A. (2020). Current application of cannabidiol (CBD) in the management and treatment of neurological disorders. Neurol. Sci..

[9] Purushothaman A., Krishnan A. (2025). Unveiling Neurological Benefits: A Review of Hemp Leaf, Flower, Seed Oil Extract, and Their Phytochemical Properties in Neurological Disorders. Cannabis Cannabinoid Res..

[10] Stockings E., Campbell G., Hall W.D., Nielsen S., Zagic D., Rahman R., Murnion B., Farrell M., Weier M., Degenhardt L. (2018). Cannabis and cannabinoids for the treatment of people with chronic noncancer pain conditions: A systematic review and meta-analysis of controlled and observational studies. Pain.

[11] Mücke M., Phillips T., Radbruch L., Petzke F., Häuser W. (2018). Cannabis-based medicines for chronic neuropathic pain in adults. Cochrane Database Syst. Rev..

[12] Wang L., Hong P.J., May C., Rehman Y., Oparin Y., Hong C.J., Hong B.Y., AminiLari M., Gallo L., Kaushal A. (2021). Medical cannabis or cannabinoids for chronic non-cancer and cancer related pain: A systematic review and meta-analysis of randomised clinical trials. BMJ.

[13] Solmi M., De Toffol M., Kim J.Y., Choi M.J., Stubbs B., Thompson T., Firth J., Miola A., Croatto G., Baggio F. (2023). Balancing risks and benefits of cannabis use: Umbrella review of meta-analyses of randomised controlled trials and observational studies. BMJ.

[14] Wang T., Collet J.P., Shapiro S., Ware M.A. (2008). Adverse effects of medical cannabinoids: A systematic review. Can. Med Assoc. J..

[15] Degenhardt L., Hall W.D. (2008). The adverse effects of cannabinoids: Implications for use of medical marijuana. Can. Med Assoc. J..

[16] Keehbauch J., Rensberry M. (2015). Effectiveness, Adverse Effects, and Safety of Medical Marijuana. Am. Fam. Physician.

[17] Cuttler C., LaFrance E.M., Craft R.M. (2022). A Large-Scale Naturalistic Examination of the Acute Effects of Cannabis on Pain. Cannabis Cannabinoid Res..

[18] AminiLari M., Kithulegoda N., Strachan P., MacKillop J., Wang L., Pallapothu S., Neumark S., Sharma S., Sethi J., Zacharias R. (2022). Benefits and Concerns regarding Use of Cannabis for Therapeutic Purposes Among People Living with Chronic Pain: A Qualitative Research Study. Pain Med..

[19] Chang-Douglass S., Mulvihill C., Pilling S. (2020). Cannabis-based medicinal products: Summary of NICE guidance. BMJ.

[20] Filippini G., Minozzi S., Borrelli F., Cinquini M., Dwan K. (2022). Cannabis and cannabinoids for symptomatic treatment for people with multiple sclerosis. Cochrane Database Syst. Rev..

[21] Tafelski S., Häuser W., Schäfer M. (2016). Efficacy, tolerability, and safety of cannabinoids for chemotherapy-induced nausea and vomiting—A systematic review of systematic reviews. Der Schmerz.

[22] Black N., Stockings E., Campbell G., Tran L.T., Zagic D., Hall W.D., Farrell M., Degenhardt L. (2019). Cannabinoids for the treatment of mental disorders and symptoms of mental disorders: A systematic review and meta-analysis. Lancet Psychiatry.

[23] Silva E.A.D.J., Medeiros W.M.B., Torro N., Sousa J.M.M., Almeida I., Costa F.B.D., Pontes K.M., Nunes E.L.G., Rosa M.D.D., Albuquerque K. (2022). Cannabis and cannabinoid use in autism spectrum disorder: A systematic review. Trends Psychiatry Psychother..

[24] Silva E.A.D.J., Medeiros W.M.B., Santos J., Sousa J.M.M., Costa F.B.D., Pontes K.M., Borges T.C., Espínola C.N.S., Andrade E.S.A.H., Nunes E.L.G. (2024). Evaluation of the efficacy and safety of cannabidiol-rich cannabis extract in children with autism spectrum disorder: Randomized, double-blind, and placebo-controlled clinical trial. Trends Psychiatry Psychother..

[25] Hoch E., Niemann D., von Keller R., Schneider M., Friemel C.M., Preuss U.W., Hasan A., Pogarell O. (2019). How effective and safe is medical cannabis as a treatment of mental disorders? A systematic review. Eur. Arch. Psychiatry Clin. Neurosci..

[26] Lim K., See Y.M., Lee J. (2017). A Systematic Review of the Effectiveness of Medical Cannabis for Psychiatric, Movement and Neurodegenerative Disorders. Clin. Psychopharmacol. Neurosci..

[27] Giorgi V., Marotto D., Batticciotto A., Atzeni F., Bongiovanni S., Sarzi-Puttini P. (2021). Cannabis and Autoimmunity: Possible Mechanisms of Action. Immunotargets Ther..

[28] Sejm R.P. (2017). Act of July 7, 2017 amending the Act on Counteracting Drug Addiction and the Act on the Reimbursement of Medicines, Foodstuffs for Particular Nutritional Purposes and Medical Devices. Dz. Ustaw.

[29] Sejm R.P. (2024). Announcement of the Minister of Health of March 7, 2024 on the announcement of the consolidated text of the Regulation of the Minister of Health on narcotic drugs, psychotropic substances, category 1 precursors and preparations containing these drugs or substances.. Dz. Ustaw.

[30] Hordowicz M., Jarosz J., Czaplińska M., Leonhard A., Klimkiewicz A. (2021). Polish Physicians’ Perspectives on Medical Cannabis Policy and Educational Needs: Results of An Online Survey. J. Clin. Med..

[31] Silczuk A., Jankowski M., Mularczyk-Tomczewska P., Olearczyk A., Baran T., Wrześniewska-Wal I., Lewandowska A., Łoś M. (2025). Public understanding of medical cannabis in Poland 7 years after legalization: Findings from a cross-sectional study. Front. Public Health.

[32] Kos M. (2025). Interpellation No. 7656 Concerning Standardized Forms of Medical Marijuana, Extraction Methods, Dosage, and Potential Adverse Effects. https://www.sejm.gov.pl/sejm10.nsf/interpelacja.xsp?typ=INT&nr=7656.

[33] Eysenbach G. (2004). Improving the quality of Web surveys: The Checklist for Reporting Results of Internet E-Surveys (CHERRIES). J. Med. Internet Res..

[34] Hordowicz M.J., Jarosz J., Klimkiewicz A., Czaplińska M., Leonhard A., Wysocka M. (2022). To Treat or Not to Treat? Polish Physicians’ Opinions about the Clinical Aspects of Cannabinoids-An Online Survey. J. Clin. Med..

[35] Oldfield K., Eathorne A., Maijers I., Beasley R., Semprini A., Braithwaite I. (2020). Knowledge and perspectives about the use of cannabis as a medicine: A mixed methods observational study in a cohort of New Zealand general practice patients. N. Z. Med. J..

[36] Abo Ziad R., Grynbaum M.B., Peleg R., Treister-Goltzman Y. (2022). The Attitudes and Beliefs of Family Physicians Regarding the Use of Medical Cannabis, Knowledge of Side Effects, and Barriers to Use: A Comparison Between Residents and Specialists. Am. J. Ther..

[37] Karanges E.A., Suraev A., Elias N., Manocha R., McGregor I.S. (2018). Knowledge and attitudes of Australian general practitioners towards medicinal cannabis: A cross-sectional survey. BMJ Open.

[38] Bawa Z., McCartney D., Manocha R., McGregor I.S. (2022). Knowledge, experiences, and attitudes of Australian General Practitioners towards medicinal cannabis: A 2021–2022 survey. BMC Prim. Care.

[39] Hordowicz M., Klimkiewicz A., Jarosz J., Wysocka M., Jastrzębska M. (2021). Knowledge, attitudes, and prescribing patterns of cannabis and cannabinoid-containing medicines among European healthcare workers: A systematic literature review. Drug Alcohol Depend..

[40] Adler L., Zacay G., Schonmann Y., Azuri J., Yehoshua I., Vinker S., Shani M., Heymann A.D., Hoffman R. (2022). Primary care physicians’ attitudes and knowledge regarding medical cannabis and willingness to prescribe it: The Israeli experience. Fam. Pract..

[41] Hazimeh B., Bou-Orm I., Mroueh M., Ammar W. (2025). Health Care Providers’ Knowledge, Attitudes, and Practices Toward Medicinal Cannabis: The Case of Lebanon. Cannabis Cannabinoid Res..

[42] Florek-Łuszczki M., Choina P., Lachowski S., Chmielewski J., Łuszczki J.J. (2025). Medical marijuana—Knowledge and opinions of primary care physicians in Lublin province, Poland. Ann. Agric. Environ. Med..

[43] Cheng K.Y.C., Harnett J.E., Davis S.R., Eassey D., Law S., Smith L. (2022). Healthcare professionals’ perspectives on the use of medicinal cannabis to manage chronic pain: A systematic search and narrative review. Pain Pract..

[44] Worster B., Ashare R.L., Hajjar E., Garber G., Smith K., Kelly E.L. (2023). Clinician Attitudes, Training, and Beliefs About Cannabis: An Interprofessional Assessment. Cannabis Cannabinoid Res..

[45] Ng J.Y., Gilotra K., Usman S., Chang Y., Busse J.W. (2021). Attitudes toward medical cannabis among family physicians practising in Ontario, Canada: A qualitative research study. CMAJ Open.

[46] Hachem Y., Abdallah S.J., Rueda S., Wiese J.L., Mehra K., Rup J., Cowan J., Vigano A., Costiniuk C.T. (2022). Healthcare practitioner perceptions on barriers impacting cannabis prescribing practices. BMC Complement. Med. Ther..

[47] Schuhmacher S., Gaid D., Bishop L.D., Fleming L., Donnan J. (2024). Planting the seeds for success: A qualitative study exploring primary healthcare providers’ perceptions about medical cannabis. PLoS ONE.

[48] Edelstein O.E. (2022). Attitudes and beliefs of medicine and social work students about medical cannabis use for epilepsy. Epilepsy Behav..

